# Influence of exposure duration on human pressure‐induced inflammatory responses: Comparison between tunnel workers and underwater divers

**DOI:** 10.14814/phy2.70130

**Published:** 2024-11-20

**Authors:** Zuha Imtiyaz, Owen J. O'Neill, Douglas Sward, Phi‐Nga Jeannie Le, Awadhesh K. Arya, Veena M. Bhopale, Abid R. Bhat, Stephen R. Thom

**Affiliations:** ^1^ Department of Emergency Medicine University of Maryland School of Medicine Baltimore Maryland USA; ^2^ US Hyperbaric Inc. Tarrytown New York USA; ^3^ Zucker School of Medicine Hofstra University Northwell Northwell New York USA; ^4^ University of Texas Medical Branch Galveston Texas USA

**Keywords:** decompression sickness, microparticles, neutrophil activation, plasma gelsolin

## Abstract

Information is scarce on human responses to high pressure exposures out of water, such as related to tunnel construction workers. We hypothesized that differences in the longer durations of exposures for tunnel workers versus underwater divers results in greater inflammatory responses linked to the pathophysiology of decompression sickness (DCS). Blood was analyzed from 15 tunnel workers (36.1 ± 10.5 (SD) years old, 6 women) exposed to 142–156 kPa pressure for 4.1–4.9 h compared to 8 SCUBA divers (39.3 ± 13.3 (SD) years old, 6 women) exposed to 149 kPa for 0.61 hours. Despite differences in pressure duration between groups, elevations were the same for blood microparticles (MPs) (128 ± 28% MPs/μl) and intra‐MPs interleukin (IL‐1β) (376 ± 212% pg/million MPs), and for decreases of plasma gelsolin (pGSN, 31 ± 27% μg/mL). The number of circulating CD66b + neutrophils and evidence of cell activation, insignificant for divers, increased in tunnel workers. Across 3 exposures, the mean neutrophil count increased 150 ± 11%. Neutrophil activation increased by 1 to 2% of cells expressing cell surface CD18, myeloperoxidase, platelet‐specific CD41, and decrease of cell bound pGSN. We conclude that MPs elevations occur rapidly in humans and reach steady state in minutes with pressure exposures and neutrophil activation requires significantly longer exposure times.

## 1 INTRODUCTION

Decompression sickness (DCS) is a condition that can occur with a rapid reduction of environmental pressure. Individuals at risk for DCS include those who return from a transient high pressure exposure while deep sea diving or work in a pressurized tunnel, and when ascending to a high altitude (Mitchell et al., 2022). Among these groups, there is a paucity of studies focused on physiological changes in pressurized tunnel workers, although they are critically important for advanced construction which typically involves massive boring machines (ONeill et al., 2024). Pressure transients can be similar between SCUBA divers and tunnel workers, but tunnel workers often spend hours under pressure versus fractions of one hour for SCUBA divers. We hypothesized that differences in the typical durations of high‐pressure exposure between these two groups would result in proportional differences in magnitude of inflammatory events linked to the pathophysiology of DCS. Thus, the focus of this project was to assess whether inflammatory responses reported in relation to SCUBA (self‐contained underwater breathing apparatus) diving differed from similar exposures to high gas pressure among tunnel workers.

DCS is traditionally viewed as arising due to gas bubble formation from insoluble gas on decompression. However, venous bubbles detected by ultrasound are inconsistently present in humans suffering DCS (Bigley et al., 2008; Francis et al., 1988; Mahon & Regis, 2014). The absence of venous gas emboli (VGE) via ultrasonography is indicative of pressure/decompression safety but among those developing DCS, high numbers of VGE are found in only ~10% of divers and 5% of those exposed to altitude (Balldin et al., 2002; Mahon & Regis, 2014). The inconsistent presence of bubbles has led to increased efforts focused instead on assessing biomarkers of inflammation (Bao et al., 2015; Bigley et al., 2008; Francis et al., 1988; Glavas et al., 2008; Martin & Thom, 2002; Pontier et al., 2012; Spisni et al., 2017; Thom et al., 2012).

Elevations of microparticles (MPs), 0.1 to 1.0 μm extracellular vesicles, occur in SCUBA divers, individuals exposed to high pressure in hyperbaric chambers and in rodent models of DCS (Barak et al., 2020; Brett et al., 2019; Madden et al., 2010; Madden, Thom, Milovanova, et al., 2014; Madden, Thom, Yang, et al., 2014; Pontier et al., 2012; Thom et al., 2011, 2012, 2018; Thom, Milovanova, et al., 2013; Vince et al., 2009). MPs initiate a systemic inflammatory response related to neutrophil activation (Thom et al., 2011; Thom, Yang, et al., 2013; Yang et al., 2012, 2013). The pathway triggering MPs formation also activates the NOD‐like receptor, pyrin containing 3 (NLRP3) inflammasome that is responsible for producing mature interleukin (IL)‐1β (Thom et al., 2014, 2017). Inflammasome assembly correlates with MP production and the MPs containing high amounts of IL‐1β are a primary factor causing diffuse vascular damage in the murine DCS model (Thom et al., 2018, 2019). When these MPs are purified and injected into naïve mice, they cause the same spectrum of injuries as seen in decompressed mice (Bhopale et al., 2021; Thom et al., 2011, 2018). Further, we have found that these inflammatory MPs formed in response to pressure have filamentous (F‐) actin on the surface. Prior work has shown that plasma gelsolin (pGSN), which depolymerizes F‐actin, is consumed in association with MPs lysis and blood pGSN levels decrease in humans exposed to pressure in a hyperbaric chamber, in open‐water SCUBA divers and in the murine DCS model (Arya, Balestra, et al., 2023; Bhopale et al., 2021; Smith et al., 1987; Yin et al., 1984).

Leukocytes are activated by high pressures of nitrogen and similar gasses via an oxidative stress process that triggers MPs production (Thom et al., 2014). These biochemical events are rapidly saturable such that in ex vivo studies of human neutrophils, maximum MPs generation occurs by 30 min. We reasoned that the differences in duration of pressure exposures between SCUBA divers and tunnel workers offered an opportunity to assess whether MPs production and neutrophil activation in vivo paralleled processes evaluated with neutrophils in ex vivo laboratory studies.

## 2 METHODS AND MATERIALS

### 2.1 Materials

Annexin‐binding buffer and the following antibodies were purchased from BD Pharmingen (San Jose, CA, USA): PE‐conjugated annexin V (cat # 556421), PE‐conjugated anti‐human CD18 (cat # 555924), APC‐conjugated anti‐human CD41a (cat # 559777), and FITC‐conjugated anti‐human CD15 (cat # 555401). BV421‐conjugated anti‐human CD66b (cat # 347201) and BV510‐conjugated anti‐human CD146 were purchased from BD Optibuild (San Jose, CA, USA cat # 747737) and BD Horizon (San Jose, CA, USA), respectively. PerCP‐Cy5.5‐conjugated anti‐human CD41a (cat # 340931), percp5.5‐conjugated anti‐human CD14 (cat # 550787), and FITC‐conjugated anti‐human myeloperoxidase (MPO, cat # 340655) were purchased from BD Biosciences (San Jose, CA, USA). AlexaFluor488‐conjugated anti‐human TMEM119 (cat # ab225494) was purchased from Abcam (Boston, MA, USA). FITC‐conjugated anti‐human thrombospondin 1 (sc‐393,504) and AF647‐conjugated anti‐human NKCC1 (cat # sc‐514,858) were purchased from Santa Cruz Biotechnology (Dallas, TX, USA).

### 2.2 Human subjects

**FIGURE 1:**
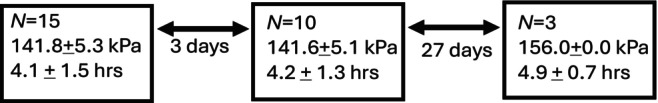
Pattern of pressure exposures for tunnel workers. The number of workers (*N*), mean pressure (kPa) and duration (hours) as well as the separation in time among the three episodes are shown. Values are mean ± SD.

All subjects gave their informed consent in writing for inclusion before they participated in the study. The study was conducted in accordance with the Declaration of Helsinki, and the protocol was approved by the University of Maryland Institutional Review Board (N^o^ HP‐00059996). Eight SCUBA divers aged 39.3 ± 13.3 years old, including 6 women participated in the study and performed a single excursion without decompression steps while performing maintenance work in large regional marine aquariums. They all wore thermal protection as wet or dry suits in water at temperatures vacillating between 24.4 and 25°C. Five were exposed for 40 minutes to 140.8 kPa and three to 162.8 kPa for 30 minutes. Thus, mean values for the 8 divers were 148.9 ± 11.1 (SD) kPa for 36.3 ± 5.2 minutes. Some results for 5 divers employed by the National Aquarium in Baltimore, MD have been reported previously (Imtiyaz et al., 2024). The project included 15 tunnel workers 36.1 ± 10.5 (SD) years old, including 6 women who were exposed to an average pressure of 148.7 kPa for about 4.5 hrs. The tunnel temperatures were 28°C and, per standard policies, tunnel workers underwent oxygen decompression at termination of their work shifts following a 100 kPa (“1.5 bar”) French decompression table. Figure 1 shows the parameters that were followed. A total of 15 workers were exposed on day #1. Three days later 10 workers were exposed on day #2, and 27 days later 3 that had been exposed on days #1 and 2 did a third exposure.

Blood from all subjects was collected in Cyto‐Chex BCT test tubes (Streck Inc., La Vista, NE, USA) following sterile, standard procedures. Blood was collected 25 mins before pressurization and 20 mins after decompression. Samples were sent by express mail to the University of Maryland laboratory where all analyses were performed following published techniques described in previous publications (Balestra et al., 2022; Thom et al., 2017). There were no adverse events identified among the divers and tunnel workers in our study.

### 2.3 MP isolation

Blood was centrifuged at 1500*g* for 5 mins, following this EDTA (final conc. 12.5 mM) was added to the supernatant to prevent MP aggregation and centrifuged at 15,000*g* for 30 mins. Supernatant containing MPs was collected and stored at 4°C until analysis by flow cytometry.

### 2.4 MP analysis

MP analysis was performed using an 8‐color, triple‐laser MACSQuant Analyzer (Miltenyi Biotech Corp., Auburn, CA, USA) and MACSQuantify software version 2.13.3. MPs were counted on the bases of size (0.3–1 μm) and surface binding of annexin V. Cells of origin were indicated by assessing expression of the surface proteins, with positive staining based on the “fluorescence minus one control test” (Tung et al., 2004).

### 2.5 Neutrophil analysis

Whole fixed blood from the Cyto‐Chex tubes (100 μL) was stained for 30 min at room temperature in the dark with optimized concentrations of antibodies listed in materials. After staining, 1 mL phosphate buffered saline (PBS) was added to dilute each sample tube prior to analysis, with the cytometer acquisition set to use anti‐human CD66b as the fluorescence trigger to recognize neutrophils. Emphasis on CD66b was based on reports indicating that the fraction of this sub‐population of neutrophils (from 6.5 to 34% in healthy human controls) increases in response to a wide variety of inflammatory stimuli (Karawajczyk et al., 2021; Opasawatchai et al., 2018; Sun et al., 2022). Neutrophil counts were assessed as CD66b‐positive cells/μl of blood and activation as % of cells expressing CD18, MPO, CD41 and pGSN.

### 2.6 Plasma gelsolin analysis

A human‐specific commercial pGSN sandwich ELISA kit (LSBio, Inc. Seattle, WA, USA, cat # LS‐F5675; with sensitivity 6.25 ng/mL and inter‐ and intra‐assay variabilities <12%) was used following the manufacturer's instructions. Serial dilutions in PBS were prepared using plasma supernatant after the 1500 g centrifugation, as described above, and analyzed concurrent with a range of known pGSN standards.

### 2.7 IL‐1β analysis

For IL‐1β analysis plasma supernatant after centrifuging the blood at 15,000*g* was taken and further centrifuged at 100,000*g* for 60 mins. After discarding the supernatant, 200 μL lysis buffer was added to the MP pellet. For the measurement of IL‐1β 20 μg of protein was used. Human‐specific ELISA kit (Invitrogen, Waltham, MA, USA, cat # 88726122; with sensitivity of 2 ng/mL) that detected pro‐ and mature forms of IL‐1β was used according to the manufacturer's instruction.

### 2.8 Statistical analysis

The results are expressed as the mean ± standard deviation (SD), and the analyses were performed using SigmaStat (v15, Jandel Scientific, San Jose, CA, USA). Data normality was assessed using the Shapiro–Wilk test. The details of the statistical analysis for each assay are provided in the figure legends. Single‐group (pre‐ and post‐activity) comparisons were evaluated by a paired *t*‐test. For multiple group comparisons, a one‐way analysis of variance (ANOVA) was used. For all the studies, the results were deemed statistically significant if *p* < 0.05.

## 3 RESULTS

### 3.1 MP analysis assay

**FIGURE 2:**
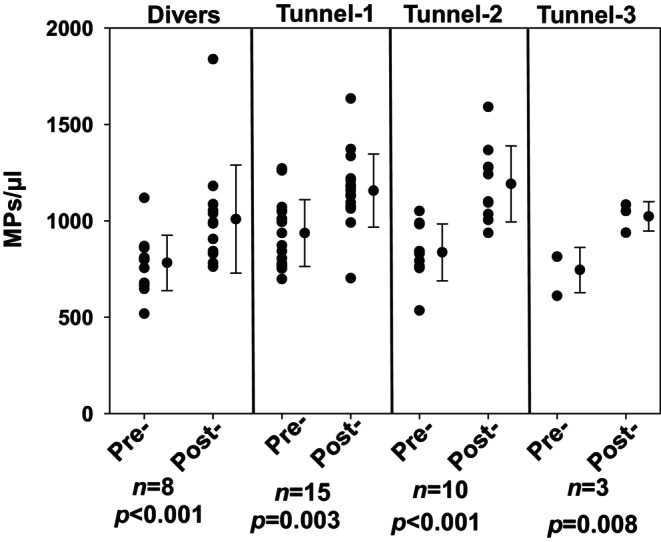
Analysis of MPs isolated from the blood of the divers and tunnel workers. Flow cytometry was used to evaluate MPs identified based on size (0.3 to 1 μm diameter) and surface expression of annexin V. Pre‐ and post‐pressure exposure values are shown for SCUBA divers and each of the exposures performed by tunnel workers as depicted in Figure 1. Individual data points, mean ± SD, number of subjects (*n*) and level of significance pre‐ versus post‐pressure [(*p*) based on *t*‐test] are shown.

**FIGURE 3:**
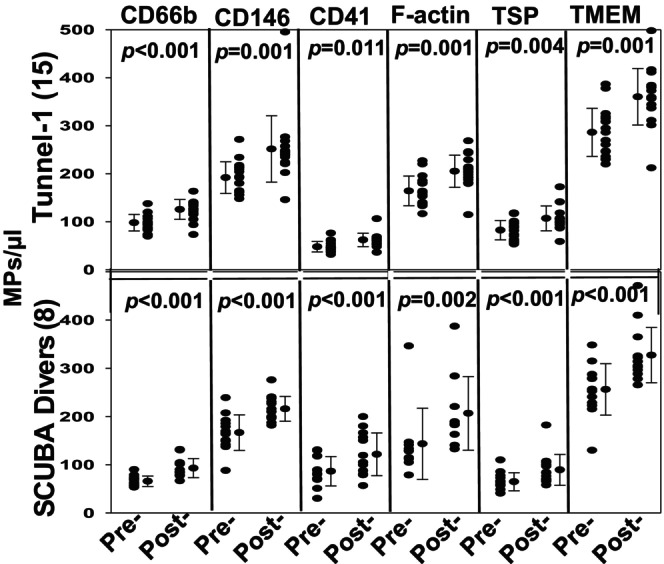
MPs subtypes in blood of divers and tunnel workers. Expression of cell‐specific proteins on the surface of MPs was evaluated by flow cytometry as described in Methods. Proteins probed on the surface of the MPs included those from neutrophils (CD66b), endothelium (CD146), platelets (CD41a), microglia (transmembrane protein 119, TMEM), and those expressing TSP‐1 and F‐Actin that was assessed by fluorescent labeled phalloidin binding. Individual data points, mean ± SD, and level of significance pre‐ versus post‐pressure [(*p*) based on *t*‐test] are shown. The number of subjects are shown on the ordinate (*N in Figure 2*). Pre‐exposure values of MPs from divers and tunnel workers were not significantly different.

Figure 2 illustrates changes in blood‐borne MPs among the SCUBA divers and in relation to the three pressure exposures by tunnel workers. There were no significant differences in responses between men and women, and the mean pre‐exposure values for all four episodes of pressurization were not significantly different (pairwise p‐values were 0.06 to 0.76, ANOVA). The levels of significance for elevations occurring in response to each exposure are as shown (Figure 2). Subsets of MPs were also analyzed. Figure 3 demonstrates changes among MPs from the first tunnel exposure and the SCUBA dive. The pre‐exposure and post‐decompression values related to tunnel 2 and 3 exposures were virtually identical to the first exposure. Proteins probed on the surface of the MPs included those from neutrophils (CD66b), endothelial cells (CD146), platelets (CD41a) and microglia (transmembrane protein 119, TMEM). We also probed for the presence of thrombospondin‐1 (TSP) and filamentous (F‐) actin, motivated by findings from prior studies (Arya, Balestra, et al., 2023; Bhat et al., 2024; Thom, Bhopale, Bhat, et al., 2023). Significant elevations were noted for all sub‐types and the magnitude of changes were similar between tunnel workers and SCUBA divers.

### 3.2 Neutrophil activation assay

CD66b + neutrophil counts did not differ pre‐ versus post‐pressurization among the SCUBA divers (533 ± 94 vs. 615 ± 181 cells/μl), but values were significantly different for the tunnel workers. The CD66b + cell count prior to the first exposure was 538 ± 141 cells/μl and post‐exposure, 767 ± 157 (*n* = 15, *p* < 0.001). Pre‐exposure values for the second and third exposures (581 ± 48 and 533 ± 94, respectively) were not significantly different from the first pre‐exposure value. However, the post‐exposure values were each significantly different from the pre‐exposure values (second exposure 803 ± 103 (*n* = 10, p < 0.001) and for the third, 726 ± 53 (*n* = 3, *p* = 0.002) cells/μl).

**FIGURE 4:**
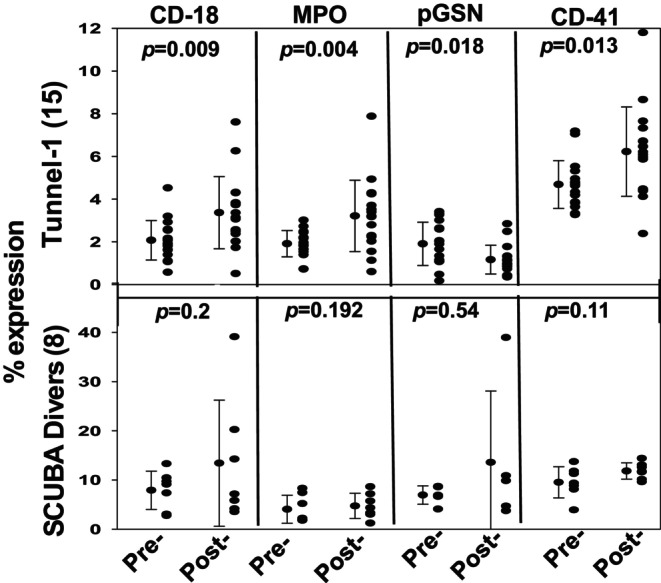
Analysis of neutrophil activation. Whole blood was used for neutrophil activation assessment by flow cytometry as described in the Methods. Neutrophils in whole blood were identified based on CD66b expression and analyzed based on the fraction of cells expressing proteins of interest, CD18, MPO, pGSN and CD41 above a threshold value were measured. Individual data points, mean ± SD, and level of significance pre‐ versus post‐pressure [(*p*) based on *t*‐test] are shown. The number of subjects is shown on the ordinate (*N in Figure 2*).

Neutrophil activation was assessed by flow cytometry as an increase in surface expression of CD18 (a component of β_2_ integrins) and myeloperoxidase (MPO), indicative of degranulation. Figure 4 illustrates responses in those involved with the first tunnel exposure and the SCUBA dives. The pre‐exposure and post‐decompression values related to tunnel 2 and 3 exposures were virtually identical to the first exposure. Significant increases occurred among the tunnel workers, but there were no significant increases in the SCUBA divers. The presence of CD41, a platelet‐specific protein, on neutrophils was also significantly increased in tunnel workers. The presence of pGSN on the neutrophil surface was motivated by recent published observations showing a decrease of pGSN in blood and on neutrophils (Bhopale et al., 2021). A statistically significant but still nominal decrease was noted among tunnel workers (expression present on 1.9 ± 1.0% pre‐pressure and 1.2 ± 0.7% post‐decompression).

### 3.3 IL‐1β in MP

**FIGURE 5:**
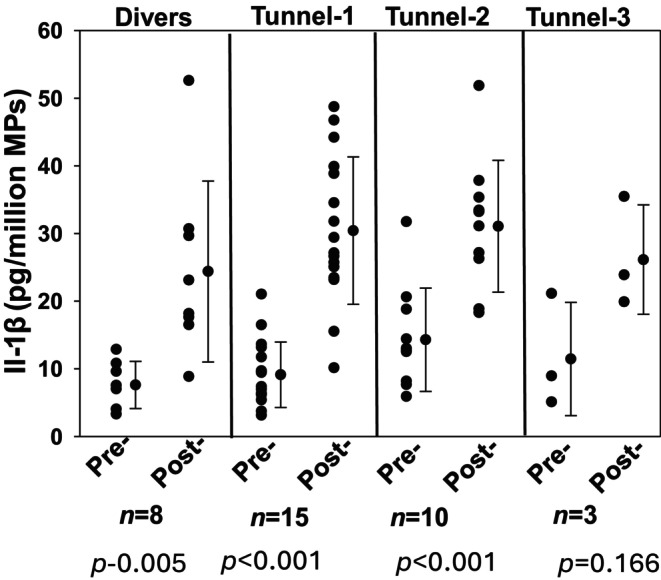
IL‐1β in MPs. Content of IL‐1β was measured in MPs by ELISA as described in Methods and expressed as pg/million MPs. Individual data points, mean ± SD, number of subjects and level of significance pre‐ versus post‐pressure [(*p*) based on *t*‐test] are shown. Pre‐exposure values of IL‐1β levels from both tunnel as well as divers show no significant difference.

Significant elevations of IL‐1β in MPs were found among SCUBA divers and the three tunnel exposures as shown in Figure 5. Once again, pre‐and post‐exposure values were similar with each of the three tunnel episodes.

### 3.4 Plasma gelsolin

**FIGURE 6:**
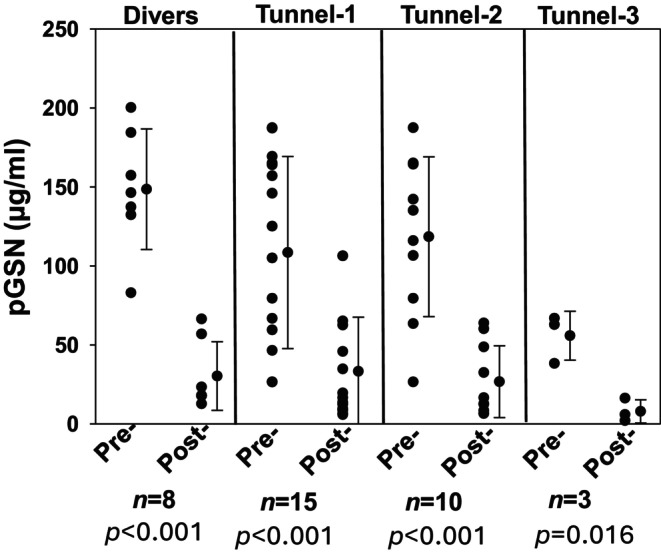
Changes in pGSN levels. Plasma gelsolin was measured by sandwich ELISA as described in Methods and expressed as μg/mL. Individual data points, mean ± SD, number of subjects and level of significance pre‐ versus post‐pressure [(*p*) based on *t*‐test] are shown. Pre‐exposure values of pGSN levels from tunnel workers and divers were not significantly different.

Plasma gelsolin (pGSN) is a cytoplasmic actin binding protein found to decrease in post‐diving samples in a murine DCS model, SCUBA divers and human subjects exposed to pressure in a hyperbaric chamber (Arya, Balestra, et al., 2023; Bhopale et al., 2021). Figure 6 shows that the pGSN levels decreased significantly with each exposure. The pre‐exposure values were not significantly different across the groups.

## 4 DISCUSSION

We conclude from this study that MPs production is comparable between pressurized tunnel workers and SCUBA divers, indicating that responses to pressure predominate over differences in activities (e.g. swimming versus working in an air atmosphere) performed while under pressure. In addition to total MPs counts, subsets expressing specific surface proteins were also comparable between tunnel workers and SCUBA divers. Note that probes for the presence of TSP‐1 and F‐actin were included because studies in the murine DCS model indicate that TSP can play a role in MPs‐mediated neutrophil activation and F‐actin expressing MPs are the sub‐set that carry inflammatory mediators such as IL‐1β (Arya, Balestra, et al., 2023;Bhat et al., 2024; Thom, Bhopale, Bhat, et al., 2023).

MPs elevations in tunnel workers were consistent across three separate exposures spanning as much as 27 days. Particle counts returned to the baseline value prior to tunnel exposures 2 and 3. This restoration of baseline and consistent elevations with pressure/decompression have also been reported for SCUBA divers performing daily excursions over a span of 6 days (Thom et al., 2015). It is notable that the MPs counts in blood were virtually the same between tunnel workers who were exposed to pressure for as long as 4.9 ± 0.7 hours (see Figure 1) and SCUBA divers exposed to pressure for just 36.3 ± 5.2 minutes. Achieving a maximum number of MPs within 36 minutes is consistent with the rate of human neutrophil MPs production ex vivo by elevated nitrogen partial pressures (Thom et al., 2014). However, whether biochemical events in vivo are saturable within 36 minutes cannot be determined as we do not know the clearance rate for the various MPs sub‐types expressing different surface proteins. Studies in the murine model indicate that F‐actin expressing MPs pose the greatest inflammatory threat and endothelial uptake may be related to the CD36 (so‐called scavenger) receptor (Bhat et al., 2024; Thom, Bhopale, Bhat, et al., 2023).

Elevations of IL‐1β following high gas pressure exposures have been described in the murine model and humans after simulated and open‐water diving (Arya, Balestra, et al., 2023; Brett et al., 2019; Thom et al., 2018, 2019). IL‐1β secretion requires unconventional pathways involving packaging into extracellular vesicles, and virtually all IL‐1β can be found within MPs generated in response to high pressure exposures (Arya, Balestra, et al., 2023; Cypryk et al., 2018). Here we show IL‐1β elevations in divers and tunnel workers are similar. This is consistent with NLRP3 inflammasome assembly occurring concurrent with MPs formation in response to high gas pressure (Thom et al., 2014). As with MPs formation itself, similar IL‐1β elevations in tunnel workers despite markedly longer pressure exposure times suggests there is a rapid arrival at steady state.

Neutrophil numbers were quantified as cells expressing CD66b and activation was assessed as an increased cell surface expression of CD18 and MPO. Elevations were identified among the tunnel workers. Absence of elevations in divers suggests that longer duration at pressure are necessary for these alterations. Note that the range of CD18 expression in tunnel workers prior to each pressurization (see Figure 4), on the order of 2% of neutrophils, was less than that for SCUBA divers (7.9 ± 3.8%, *p* < 0.002, ANOVA). This higher baseline level of neutrophil activation among divers was not observed in a prior publication involving professional saturation divers, where pre‐diving values were similar to those of the tunnel workers for both CD18 and MPO (Arya, Balestra, et al., 2023). The reason for the differences and whether they pose physiological significance are not known. We speculate that frequent prior pressure exposures in the professional saturation divers and perhaps exposure to higher pressures (as much as ~1113 kPa) may have resulted in a down‐regulation of baseline surface β_2_ integrin expression and propensity for degranulation. Platelet CD41 protein on neutrophils was also significantly increased in tunnel workers (Figure 4). Studies have shown that activated platelets shed MPs and that platelets and their MPs can adhere to neutrophils, which could exacerbate neutrophil activation (Jy et al., 1995; Thom et al., 2011; Valadez‐Cosmes et al., 2021). While this was not identified in SCUBA divers in the current study, it has been reported when divers were exposed to higher pressures (Thom et al., 2012; Thom, Milovanova, et al., 2013).

Gelsolin is an actin‐depolymerizing protein, and its extracellular isoform is plasma gelsolin (pGSN) (Smith et al., 1987; Yin et al., 1984). A decrease in the levels of pGSN has been reported in various traumatic injuries and pathological conditions (Suhler et al., 1997; Thom, Bhopale, Arya, et al., 2023; Xu et al., 2012). In this study, we observed that pGSN in blood of tunnel workers was significantly decreased after each exposure in a similar pattern to the SCUBA divers. These changes are consistent with previous studies (Arya, Balestra, et al., 2023; Bhopale et al., 2021). Loss of pGSN appears to be related to binding and lysis of F‐actin expressing MPs but may also arise due to protein binding to F‐actin liberated from cell destruction followed by proteolysis.

The magnitude of MPs elevations seen among SCUBA divers and tunnel workers in this study was comparable to elevations reported for divers exposed to pressures ranging from 111 to 1151 kPa (Arya, Balestra, et al., 2023; Thom et al., 2015). This similarity is contrary to the direct dose‐dependency between MPs production and gas partial pressures seen in the murine model and ex vivo studies of human neutrophils (Thom et al., 2011, 2014). This could occur if MPs clearance is more rapid in humans, or if production mechanisms differ. In addition to a direct effect of gas pressure stimulating neutrophil MPs production, multiple generations of MPs can be produced by a cascading process. We recently showed that provocative pressure/decompression and also exposure to carbon monoxide can trigger MPs production in the brain, and after gaining access to the blood stream these MPs activate neutrophils to produce a new generation of MPs that cause neuroinflammation in a cyclic process persisting for weeks (Bhat et al., 2024; Thom, Bhopale, Bhat, et al., 2023).

Limitations in our study include the relatively small sample size, although data are consistent among the individual subjects. We did not obtain serial blood samples over many hours to days. Among SCUBA divers and research subjects exposed to pressure in a hyperbaric chamber, peak MPs levels and neutrophil activation parameters typically occur at ~2 hours and resolve within 24 hours, but whether prolonged exposures among tunnel workers may have resulted in a different pattern is unknown (Arya, Balestra, et al., 2023; Brett et al., 2019; Thom et al., 2015). Similarly, we cannot discern whether the moderate differences in environmental temperature or the possibility of air contaminants in the tunnels may have influenced results. It also should be noted that the tunnel workers underwent oxygen decompression on termination of their work shifts as a safety measure. Oxygen pressurization/decompression has not been found to trigger MPs mediated inflammatory responses in murine studies (Yang et al., 1985). Moderate abatement of MPs responses has been observed in divers using gas mixtures enriched with oxygen, and human investigations currently underway suggest that hyperoxia diminishes MPs production (preliminary unpublished observations) (Arya, Balestra, et al., 2023). These findings imply that neutrophil activation results found among tunnel workers may be lower than if samples had been obtained prior to decompression procedures. Further work is needed to clarify this issue. The data do not allow conjecture on whether the pattern of pressure exposure with longer durations pose a greater DCS threat to tunnel workers than is observed for SCUBA divers.

## AUTHOR CONTRIBUTIONS

Z.I., O.J.O., and S.R.T. conceived and designed the research. O.J.O., D.S., and P.J.L. enrolled and obtained blood samples from study subjects. Z.I., A.K.A., V.M.B., A.R.B., and S.R.T. conducted laboratory analysis. The experiments. Z.I., A.K.A., A.R.B., and S.R.T. interpreted results. Z.I. and S.R.T. drafted the manuscript, S.R.T. prepared figures. All authors assisted with editing the manuscript and approved the final version.

## FUNDING INFORMATION

This work was supported by a grant N00014‐22‐1‐2818 from the U.S. Office of Naval Research and R01‐NS‐122855 from National Institute of Neurological Disorders and Stroke.

## CONFLICT OF INTEREST STATEMENT

The authors declare no conflicts of interest.

## Data Availability

Data are available at request from the authors.
